# Morphological Diversity of Calretinin Interneurons Generated From Adult Mouse Olfactory Bulb Core Neural Stem Cells

**DOI:** 10.3389/fcell.2022.932297

**Published:** 2022-06-29

**Authors:** Francisco J. Fernández Acosta, Inma Luque-Molina, Rebeca Vecino, Eva Díaz-Guerra, Çagla Defterali, Jaime Pignatelli, Carlos Vicario

**Affiliations:** ^1^ Instituto Cajal (IC), CSIC, Madrid, Spain; ^2^ Centro de Investigación Biomédica en Red Sobre Enfermedades Neurodegenerativas (CIBERNED), Instituto de Salud Carlos III (ISCIII), Madrid, Spain

**Keywords:** adult neurogenesis, calretinin neuron subtypes, morphology, olfactory bulb layers, 3D analysis, transcription factors

## Abstract

Neural stem cells (NSCs) in the olfactory bulb (OB) core can generate mature interneurons in the adult mice brain. The vast majority of these adult generated cells express the calcium-binding protein Calretinin (CalR), and they migrate towards different OB layers. However, these cells have yet to be fully characterized and hence, to achieve this we injected retroviral particles expressing GFP into the OB core of adult animals and found that the CalR^+^ neurons generated from NSCs mainly migrate to the granule cell layer (GCL) and glomerular layer (GL) in similar proportions. In addition, since morphology and function are closely related, we used three-dimensional imaging techniques to analyze the morphology of these adult born cells, describing new subtypes of CalR^+^ interneurons based on their dendritic arborizations and projections, as well as their localization in the GCL or GL. We also show that the migration and morphology of these newly generated neurons can be altered by misexpressing the transcription factor Tbr1 in the OB core. Therefore, the morphology acquired by neurons located in a specific OB layer is the result of a combination of both extrinsic (e.g., layer allocation) and intrinsic mechanisms (e.g., transcription factors). Defining the cellular processes and molecular mechanisms that govern adult neurogenesis might help better understand brain circuit formation and plasticity, as well as eventually opening the way to develop strategies for brain repair.

## Introduction

New neurons continue to be generated from neural stem cells (NSCs) present in the adult brain (Altman and Das, 1965; Bayer, 1983; Bond et al., 2015). This process, known as adult neurogenesis, takes place in restricted areas of the CNS in higher and lower vertebrates (Lledo et al., 2006). Although the presence of adult neurogenesis in humans remains to be confirmed (Alvarez-Buylla et al., 2022; Arellano et al., 2022; Terreros-Roncal et al., 2022), understanding the mechanisms that mediate NSC activation and proliferation, as well as the events that drive the differentiation, migration and integration of new neurons, represents a powerful tool that might help us to promote brain repair following traumatic, cardiovascular or neurodegenerative insults (Nano and Bhaduri, 2022). To date, research in this field has mainly relied on rodent models, leading to the extensive characterization of NSCs in their subgranular (SGZ) and subventricular zone (SVZ: Ming and Song, 2011; Obernier and Alvarez-Buylla, 2019).

Besides these well-known neurogenic niches, there is compelling evidence suggesting the presence of endogenous NSCs in the elbow of the rostral migratory stream (RMS: Hack et al., 2005; Alonso et al., 2008; Mendoza-Torreblanca et al., 2008; Schweyer et al., 2019) and in the olfactory bulb (OB) itself (Fukushima et al., 2002; Gritti et al., 2002; Liu and Martin, 2003; Giachino and Taylor, 2009; Vergaño-Vera et al., 2009; Moreno-Estellés et al., 2012; Defterali et al., 2016). Supporting this concept, by injecting retroviral particles into the SVZ and analyzing the OB core, we previously found that almost none (1–2%) of the SVZ-derived cells proliferate in the OB core. Although we cannot completely rule out the possibility that there is a small proportion of cells of mixed origin, our results indicate that the vast majority of dividing cells detected in the OB core (and therefore targeted with the retroviral particles) are not derived from the SVZ (Defteralı et al., 2021). In fact, OB core NSCs were seen to generate neurons and glia *in vivo*, confirming their multipotency. The majority of these adult-born neurons express the calcium-binding protein Calretinin (CalR), a common marker of OB interneurons, and they were able to be incorporated into OB circuits (Defteralı et al., 2021). In addition, there is evidence of NSCs in the human OB (Pagano et al., 2000; Liu and Martin, 2003; Bédard and Parent, 2004; Marei and Ahmed, 2013; Marei et al., 2018). Therefore, it would be of interest to determine the role of each NSC population in the shaping of the adult OB, assessing what type of neurons emerge and how their generation is regulated. Ultimately, understanding the full range of interneurons born postnatally will help us understand the capacity and role of adult neurogenesis (Merkle et al., 2014).

Distinct interneuron subtypes arise from different domains in the adult SVZ in a defined spatial pattern, giving rise to neuroblasts that migrate through the RMS towards the OB where they integrate into the granule cell layer (GCL), the glomerular layer (GL) and less frequently, the external plexiform layer (EPL: Lledo et al., 2008; Yang, 2008). More precisely, NSCs located in the rostral and medial/dorsal aspects of the SVZ generate neurons that populate more superficial layers of the OB, while caudal and lateral NSCs are correlated with interneurons located in deeper layers (Fiorelli et al., 2015). Granule cells/neurons (GCs) are the main type of interneuron that experience turnover in adulthood (Petreanu and Alvarez-Buylla, 2002). These cells all express the inhibitory marker GABA, and they can be classified into at least 7 different subpopulations based on the location of their cell body (deep, superficial GCL or in the mitral cell layer -MCL), their connectivity and their expression of CalR (Lim and Alvarez-Buylla, 2014). Furthermore, in the GL there are three chemically defined non-overlapping subtypes of periglomerular cells/neurons (PGCs) based on their expression of CalR, calbindin (CalB) and tyrosine hydroxylase (TH: Kosaka and Kosaka, 2007, 2011; Hurtado-Chong et al., 2009). Depending on their connectivity, they can also be distinguished as type 1 PGCs (TH^+^) that receive direct synaptic inputs from olfactory nerve (ON) terminals and type 2 PGCs (CalR^+^ or CalB^+^) that do not contact the ON but rather, that receive inputs from mitral and tufted cells (Kiyokage et al., 2010). Finally, interneurons in the EPL express parvalbumin (PV) and the vasoactive intestinal polypeptide (VIP: Yang, 2008; Merkle et al., 2014), and they contribute to the perisomatic inhibition of principal cells (Crespo et al., 2013).

Due to the relationship between morphology and function, we performed rigorous morphological analyses in order to assess the diversity of CalR^+^ interneurons locally generated in the OB, given that they represent the most numerous population of cells derived from adult OB core NSCs (Defteralı et al., 2021). We describe diverse neuronal subtypes in the GCL and GL, characterized by the number, length and branching of their dendrites, as well as the layers where they could potentially establish synaptic contacts. We also show that this neurogenic process can be affected by overexpression of the transcription factor Tbr1 in the adult OB core.

## Materials and Methods

### Animals

All animal care and handling was carried out in accordance with European Union guidelines (directive 2010/63/EU) and Spanish national legislation (Law 32/2007 and RD 53/2013). All the protocols were approved by the Ethics Committees of the Consejo Superior de Investigaciones Científicas (CSIC, Madrid) and the Comunidad de Madrid (Spain). Food and water were administered *ad libitum*, and the environmental conditions were strictly controlled: 12 h light/dark cycle, 22°C temperature and 44% humidity. All efforts were made to ameliorate animal suffering.

### Generation of Plasmids Expressing a Human Tbr1 and Production of Retroviral Particles

Stable Tbr1 expression was achieved using a modified Moloney murine leukaemia virus-based retroviral vector carrying a CMV (Otaegi et al., 2006; Méndez-Gómez et al., 2011) or a CAG promoter and a WPRE sequence (Supplementary Figure S1 **)**. The retroviral construct pRV-IRES-EGFP (simply referred to as the EGFP vector) was used as a control in infection experiments. A construct encoding the complete human Tbr1 cDNA (2.7 kb) with the open reading frame (ORF) and the 5′ and 3′ untranslated region (UTR) was excised from a pBSK plasmid (Bulfone et al., 1995) using XbaI and cloned into the pRV-IRES-EGFP vector. In the resulting pRV–Tbr1-IRES-EGFP vector (Tbr1-EGFP vector), the *Tbr1* insert was sequenced and corresponded to the expected fragment in a 5′ to 3′ orientation. A second Tbr1-EGFP vector was constructed by PCR cloning of the ORF of Tbr1, using specific 5′ and 3′ primers containing XhoI and EcoRI restriction sites (sense primer: 5′-GTT​CTC​GAG​CTA​TGC​AGC​TGG​AGC​ACT​GCC-3'; antisense primer: 5′-GCA​CTT​AAG​GCC​TAG​CTG​TGC​GAG​TAG​AAG​CC-3′). The amplified PCR fragment was first cloned into a TOPO vector (TOPO TA cloning, Invitrogen) and a clone containing no coding mutations was selected. The *Tbr1* fragment from this clone was ligated into the pRV-IRES-EGFP vector and then sequenced (Méndez-Gómez et al., 2011). The vector containing the *Tbr1* ORF was used in the present study. The CAG promoter was removed from the pCAGGS plasmid (kindly provided by Dr Jun-ichi-Miyazaki, Osaka University, Japan (Niwa et al., 1991): with the SalI and XhoI restriction enzymes, and it was then cloned upstream of the human *Tbr1 ORF* or *IRES* in the XhoI digested pRV-hTbr1-IRES-EGFP and pRV-IRES-EGFP plasmids, respectively. The WPRE region was cloned from a pLV-IRES-EGFP-WPRE vector (a kind gift of Dr Pantelis Tsoulfas, The Miami Project to Cure Paralysis, Miami, United States) by digesting it with SalI and EcoRI, and it was inserted into the SalI digested pCAG-Tbr1-IRES-EGFP and pCAG-IRES-EGFP plasmids. After transforming bacteria with the corresponding plasmids and extracting the DNA, a Tbr1 clone was selected free of mutations in the Tbr1 coding region (Supplementary Figure S1). The two plasmids (pCAG-Tbr1-EGFP and pCAG-EGFP) were transfected into 293T-HEK cells to confirm the expression of both Tbr1 and EGFP, analyzed by immunostaining with primary antibodies against GFP (1:1000, rat: Nacalai Tesque 04404-84, RRID:AB_10013361) and Tbr1 (1:1000, rabbit: Abcam AB31940, RRID:AB_2200219), both detected with fluorescent secondary antibodies The CMV and CAG plasmids were used to produce retroviral particles expressing EGFP (pEGFP vector) or Tbr1-EGFP (pTbr1-EGFP vector), which were produced and titered using our published procedures (Méndez-Gómez et al., 2011; Vergaño-Vera et al., 2015). In brief, viral particles were obtained by transfecting 293T HEK cells with the corresponding plasmids; then the supernatants were collected and concentrated by ultracentrifugation, and the resulting titers were in the range of 10^7^ IU/ml (as measured by infecting NIH 3T3 cells with increasing volumes of supernatant followed by flow cytometry analysis). Retroviral particles were also obtained by transfecting the retroviral plasmids into 1F8 cells, a monoclonal cell line derived from a stock of 293GPG [which expresses the vesicular stomatitis virus G (VSV-G) protein and the Gag-Pol (GP) polyprotein] cells (Heinrich et al., 2011). The supernatant containing the viral particles was concentrated by ultracentrifugation and the resulting titers were in the range of 10^9^−2 × 10^11^ colony forming units (cfu)/ml. Similar results were obtained using both CMV and CAG plasmids and therefore, they were combined in the analysis.

### Injection of Retroviral Particles Into the Adult Olfactory Bulb, Perfusion and Immunohistochemistry

Unilateral injections were performed on nine-week-old wild-type C57BL/6N female mice using a digital stereotaxic apparatus, as reported previously (Defteralı et al., 2021). The animals were anesthetized with an intraperitoneal (ip) pentobarbital on day 21 post injection (21 dpi) and then perfused transcardially with 0.9% NaCl followed by 4% paraformaldehyde (PFA). For the Tbr1 study, both OBs of the same animal (9-weeks-old) were injected: one receiving retroviral particles expressing the pEGFP vector and the other retroviral particles expressing the pTbr1-EGFP vector. Animals were injected and perfused at 7 dpi, as described above. For all injections, a total volume of 2 µl was injected at the coordinates: anteroposterior to bregma +5.1 mm, lateral to midline +0.8 mm, ventral to dura −1.1 mm (Supplementary Figure S2A).

The animal’s brain was post-fixed for 48 h, embedded in 3% agarose and 40 µm vibratome sections were processed for immunohistochemistry. Sections were permeabilized in a solution containing 0.4% Triton X-100 and 10% normal serum, and then incubated overnight at room temperature (RT) with primary antibodies against GFP (1:750, rat: Nacalai Tesque Cat# 04404-84, RRID:AB_10013361) and Tbr1 (1:1000, rabbit: Abcam AB31940, RRID:AB_2200219) or GFP and CalR (1:3000, mouse: Swant Cat# 010399, RRID:AB_2313763) (Hurtado-Chong et al., 2009; Méndez-Gómez et al., 2011; Defterali et al., 2021). Subsequently, the samples were washed with PBS and incubated for 4 h at RT in the dark with the corresponding secondary antibody: Alexa Fluor 488 anti-rat IgG (1:750, donkey, Invitrogen Cat# A21208), Alexa Fluor 594 anti-rabbit IgG (1:1000, donkey, Molecular Probes Cat# A11012) and Alexa Fluor 594 anti-mouse IgG (1:750, donkey, Invitrogen Cat# A21203). The sections were then washed, exposed to Hoechst (1 μg/ml: ThermoFisher Cat# H3570) and mounted on glass microscope slides in Mowiol. Controls were performed to confirm the specificity of the antibodies. In particular, The Tbr1 antibody specifically labels mitral neurons and some tufted neurons in the OB (both glutamatergic), as well as glutamatergic neurons in the cerebral cortex and the hippocampus (Hevner et al., 2001; Hurtado-Chong et al., 2009; Méndez-Gómez et al., 2011). Negative controls were performed in each experiment and no specific signal was detected in the presence of the primary antibodies and in the absence of the secondary antibodies.

### Confocal Imaging and Morphological Analysis of GFP^+^/CalR^+^ Cells

Confocal images (Leica TCS SP-5) of all double-labeled (GFP^+^ and CalR^+^) cells were captured from the injected hemisphere of each section along the rostro-caudal axis (32 sections in total, from 3 different animals). To capture the whole cell, images were taken at a resolution of 1024 × 1024 in the z-plane every 1–2 μm using a ×63 oil-immersion objective. After analyzing the co-localization of GFP and CalR, a total of 77 double labelled cells were further characterized. The neurons selected had unambiguous staining for both markers and one or more primary process emanating from the cell body.

Morphological analysis was performed using the Imaris software (Bitplane, v 9.0.2), which produced a three-dimensional image of each cell. Thus, the cell body was reconstructed using the “Surfaces” tool, defining a region of interest (ROI) around the soma and setting the surface detail at 0.481 µm. For the dendrites, the “Filaments” tool was used so that the AutoPath algorithm could trace each dendritic process and their branches from a starting point at the center of the cell body. Both the cell body and the dendrites were reconstructed using the green channel (GFP) as the source channel (Supplementary Video S1), and then analyzed. All processes and branches were labeled as dendrites since no axonal-like processes were observed (granule cells do not have an axon: Lledo et al., 2008) and the GFP staining was not strong enough to label the axons of short-axon glomerular neurons (SACs). The localization of each cell in a particular OB layer, the shape of their cell body, and the volume, number and length of the primary processes (those emanating from the cell body), and of the secondary, tertiary and quaternary dendrite branches were recorded. The total dendrite length, and total number of branch and terminal points were also measured. Moreover, a Sholl analysis was performed by placing concentric circles around the center of the cell body with radial increments of 2 μm.

In the Tbr1 study, we manually counted every EGFP^+^ cell detected in serial confocal microscope sections of four OBs from six mice per condition. The images were taken from the whole Z-stack using ImageJ and the impact of Tbr1 overexpression on the morphology of GCs and on PGCs was also evaluated using ImageJ software (NIH, Bethesda, MD), analyzing the following parameters from 20 randomly selected neurons: total length, cell body perimeter and the number of primary, secondary and tertiary dendrites.

### Statistical Analysis

The data is shown as the mean ± SEM. The statistical methods used in each experiment are indicated in the Results and the figure legends. For the morphological analysis, an unpaired two-tailed Student’s t-test was used to compare two experimental conditions, with Welch’s correction applied when the F test indicated significant differences between the variances. One-way ANOVA with a Tukey’s post hoc multiple comparison test was performed when comparing more than two groups. In both instances, normality (Gaussian distribution) was assessed with a Kolmogorov-Smirnov test and equal variances were measured by Bartlett´s test. When the data didn’t follow a Gaussian distribution, a non-parametric Mann-Whitney U-test or Kruskal-Wallis test was applied with a post-hoc Dunn’s test*.* For the Sholl analysis, a two-way ANOVA followed by Bonferroni’s multiple comparisons test was used. Statistical significance was set at *p* < 0.05 and GraphPad Prism 8.0 was chosen for all statistical analyses.

## Results

### Olfactory Bulb Core Neural Stem Cells Give Rise Mainly to Granule Cells and Periglomerular Cells *in vivo*

**FIGURE 1:**
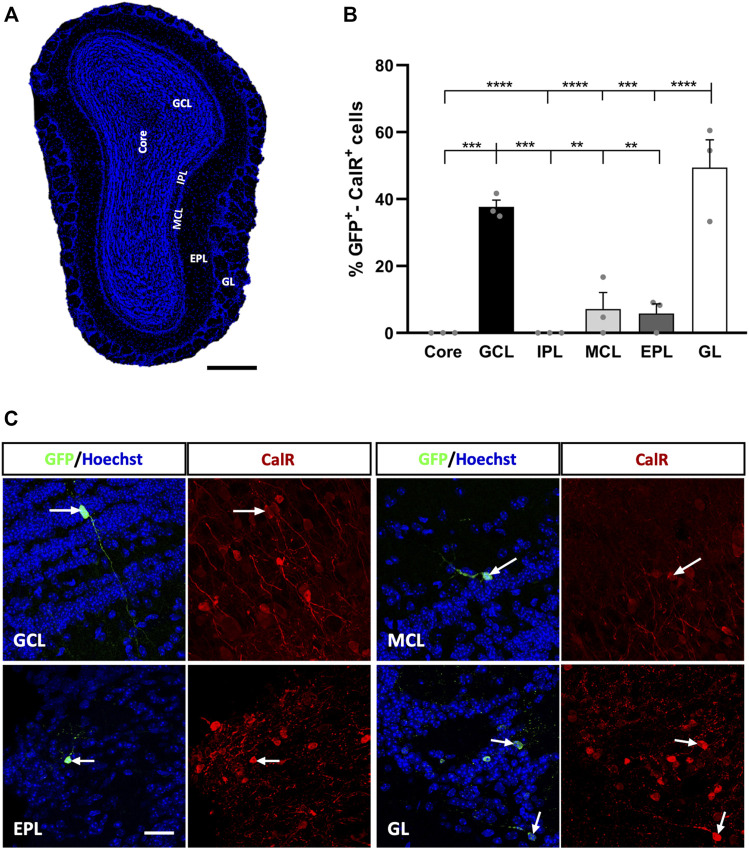
Calretinin interneurons derived from adult OB core-EGFP^+^ NSCs migrate from the core to different neuronal layers at 21 days post-injection (dpi). **(A)** Coronal section of the OB stained with Hoechst to show its layers: OB core; GCL, granule cell layer; GL, glomerular layer; IPL, internal plexiform layer; MCL, mitral cell layer; EPL, external plexiform layer. **(B)** Quantification of GFP^+^-CalR^+^ cells in the different layers of the OB. Adult-born CalR^+^ neurons migrated preferentially towards the GCL (37.67%) and GL (49.43%), while the remaining layers received few neurons (MCL, 7.13%; EPL, 5.8%) or no cells at all (Core and IPL: ^**^ *p* < 0.01, ^***^ *p* < 0.001, ^****^ *p* < 0.0001; one-way ANOVA followed by Tukey´s post hoc test). **(C)** Representative images of neurons in the GCL, MCL, EPL, and GL immunostained for GFP (green) and CalR (red). The bars show the mean ± SEM (*n* = 3 animals). Scale bars in **(A)** = 350 μm, **(C)** = 25 µm.

CalR neurons represent the largest population of newly generated cells from adult OB core NSCs (Defteralı et al., 2021) and thus, we carried out a complete morphological analysis in order to define all the possible neuronal subtypes. To describe this population, retroviral particles expressing EGFP were injected into the adult mouse OB core, and sections were analysed at 21 dpi. These sections were then immunostained with antibodies against GFP (to label adult-born cells) and CalR, and the distribution of the double-labeled cells in the different OB layers was determined (Figures 1A,B).

We observed preferential migration of GFP^+^-CalR^+^ neurons towards the GL (49.43% of these cells) and GCL (37.67%), while the percentage of labelled cells reaching the rest of the OB layers was quite low or undetectable (Figure 1B). Hence, the MCL received 7.13% of the GFP^+^-CalR^+^ neurons (^***^ *p* = 0.0001 vs. GL; ^**^ *p* = 0.0025 vs. GCL, one-way ANOVA followed by Tukey’s post hoc test) and the EPL 5.8% (^****^ *p* < 0.0001 vs. GL, ^**^ *p* = 0.0018 vs. GCL; one-way ANOVA with Tukey’s post hoc test), whereas no double-labeled cells were found in the OB core (^****^ *p* < 0.0001 vs. GL, ^***^ *p* = 0.0004 vs. GCL; one-way ANOVA with Tukey’s post-test) or the IPL (^****^ *p* < 0.0001 vs. GL, ^***^ *p* = 0.0004 vs. GCL; one-way ANOVA with Tukey’s post-test). Moreover, the adult-born CalR^+^ neurons migrating to the different layers adopted different morphologies (Figure 1C), which led us to perform a more in-depth characterization of these cells to quantify these differences.

### Morphological Diversity of Adult-Born CalR^+^ Interneurons is Influenced by the Layer to Which They Migrate

**FIGURE 2:**
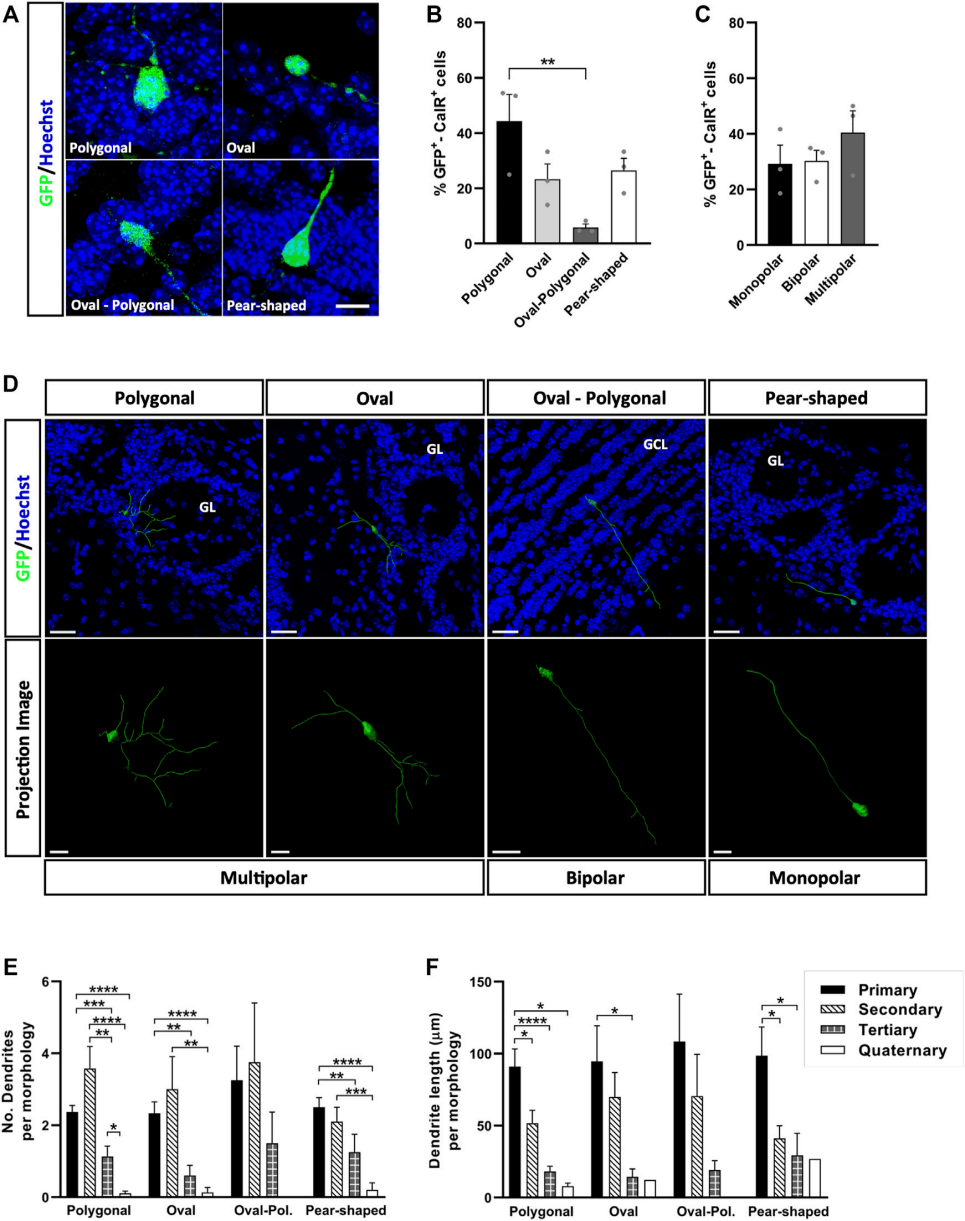
Cell body shape doesn’t affect the patterns of dendrite branching of newly generated CalR interneurons in the adult OB. **(A)** Immunohistochemical staining for GFP (green) revealed the four different shapes described. **(B)** The majority of the cells had a polygonal soma (44.33%), which was significantly more common than oval-polygonal shape (5.83%, ^**^ *p* = 0.0085; one-way ANOVA followed by Tukey’s post hoc test). Oval and pear-shaped cells accounted for 23.33 and 26.47% of the total, respectively. **(C)** The number of processes arising from the soma was also analyzed (one, two or three and more), yet no significant differences were found between monopolar, bipolar and multipolar GFP^+^-CalR^+^ neurons. **(D)** Representative 3D reconstructions of several neurons with a different shaped cell body and dendrite morphologies, which can be seen integrated in the tissue (histochemical images, top) or isolated on a black background for better detail (bottom images). All dendrites were hierarchically stratified, and their number **(E)** and length **(F)** were compared between groups. Although no significant differences were found in the number and length of dendrites as a function of the different cell body shapes, changes were evident within each group whereby secondary dendrites were generally more abundant, yet primary dendrites were the longest. No quaternary dendrites were found in oval-polygonal cells (^*^ *p* < 0.05, ^**^ *p* < 0.01, ^***^ *p* < 0.001, ^****^ *p* < 0.0001; Kruskal-Wallis with Dunn’s post hoc test). The bars represent the mean ± SEM: *n* = 3 animals **(B,C)**; *n* = 38 (Polygonal), 15 (Oval), 4 (Oval-polygonal), 20 (Pear-shaped) cells **(E,F)**. Scale bars in **(A)** = 10 μm **(D)** = 30 µm (top images), 10 µm (bottom images: multipolar and monopolar neurons), 20 µm (bottom image: bipolar neuron). CalR, calretinin; GCL, granule cell layer; GL, glomerular layer.

To carry out this morphological analysis, we first obtained three-dimensional reconstructions for each GFP^+^-CalR^+^ cell (Supplementary Video S1), which provided us with more complete and reliable information than conventional (two-dimensional) confocal images. In this way, we distinguished four different cell body shapes: polygonal, oval, oval-polygonal and pear-shaped (Figure 2A). Cells with a polygonal morphology (44.33%) were significantly more abundant than those with an oval-polygonal morphology (5.83% ^**^ *p* = 0.0085; one-way ANOVA followed by Tukey’s post hoc test), whereas oval and pear-shaped cells accounted for 23.33 and 26.47% of the cells, respectively (Figure 2B). We also counted the number of primary dendrites emerging from the soma of the cells (one, two or three or more), but no differences were found between monopolar, bipolar and multipolar neurons (Figure 2C). An example of each cell body shape and the number of primary processes is shown in Figure 2D, where the projected neurons are portrayed within the tissue (upper panels) or in isolation (lower panels).

The combination of different-order dendrites with varying lengths offers unique ways to occupy space, which could translate into a specific ways of interacting with existing circuits. Thus, it is important to identify factors that may regulate these processes. Since we detected variability in the ramifications between the different groups of neurons (Figure 2D), we analyzed the number (Figure 2E) and length (Figure 2F) of the dendrites associated with each cell body shape, considering the dendrites hierarchically, from primary (those emerging from the soma) to quaternary (the highest branching order identified). Secondary dendrites were the most numerous for the neurons with a polygonal (^**^ *p* = 0.0011 vs. tertiary, ^****^ *p* < 0.0001 vs. quaternary; Kruskal-Wallis with Dunn’s post hoc test) or oval cell body (^**^ *p* = 0.0056 vs. quaternary; Kruskal-Wallis with Dunn’s post-test), followed by primary and tertiary dendrites (Figure 2E). No differences were found in the dendritic branching of neurons with an oval-polygonal cell body, although these cells were the scarcest and the only neurons without quaternary dendrites. For pear-shaped cells, primary and secondary dendrites were the most abundant, followed by tertiary (^**^ *p* = 0.0057 vs. primary; Kruskal-Wallis followed by Dunn’s post hoc test) and quaternary ones (^****^ *p* < 0.0001 vs. primary, ^***^ *p* = 0.0002 vs. secondary; Kruskal-Wallis with Dunn’s post-test). In terms of length (Figure 2F), primary dendrites were the longest for each type of cell body described, except for oval-polygonal (polygonal ^*^ *p* = 0.0191 vs. secondary, ^****^ *p* < 0.0001 vs. tertiary, ^*^ *p* = 0.0107 vs. quaternary; oval ^*^ *p* = 0.0232 vs. tertiary; pear-shaped ^*^ *p* = 0.0246 vs. secondary, ^*^ *p* = 0.0288 vs. tertiary: Kruskal-Wallis with Dunn’s post hoc test), followed by secondary and tertiary dendrites. However, no significant differences were found between each type of cell body when comparing dendrite morphologies in terms of both number and length. Together, these results show that the pattern of dendrite branching of CalR^+^ neurons and their growth across the OB layers does not seem to be influenced by cell body shape.

**FIGURE 3:**
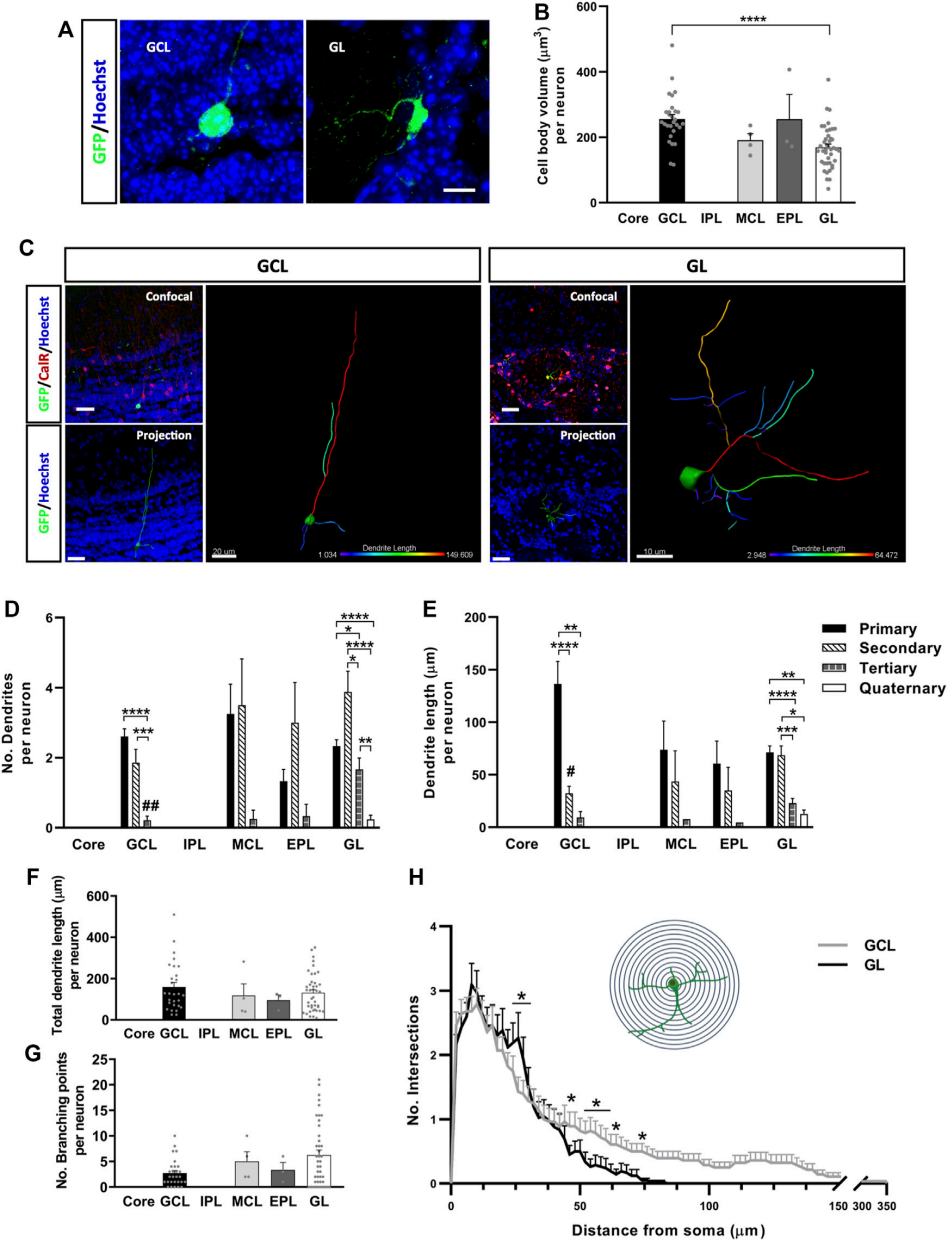
Calretinin neurons derived from adult OB core NSCs have different dendrite morphologies depending on their location in the OB layers. Representative images **(A)** and quantification **(B)** of the cell body volume of GFP^+^ neurons in the different layers of the OB. Neurons in the GCL had a significantly larger soma volume than those in the GL (^****^ *p* < 0.0001; Kruskal-Wallis with Dunn’s post hoc test). **(C)** Representative images and 3D projections of the different morphologies displayed by cells located in the GCL and GL. When compared to GL neurons, GCL neurons had fewer **(D)** but longer dendrites overall **(E)**. The projection image is color-coded for dendrite length and branching. **(D)** Quantification of the number of dendrites per neuron. Cells from the GCL had more primary and secondary dendrites (^***^ *p* < 0.001, ^****^ *p* < 0.0001; Kruskal-Wallis with Dunn’s post hoc test) than tertiary ones, which in turn were significantly less numerous relative to those in GL cells (^##^ *p* = 0.014; Kruskal-Wallis followed by Dunn’s post hoc test). Neurons located in the GL had significantly more secondary dendrites (^*^ *p* = 0.028 vs. tertiary, ^****^ *p* < 0.0001 vs. quaternary; Kruskal-Wallis with Dunn’s post hoc test), and they were the only group to present quaternary dendrites. No significant changes were detected in neurons located in the MCL and EPL. **(E)** Quantification of the dendrite length per neuron. GCL cells had the longest primary dendrites (**p = 0.010, ****p < 0.0001; Kruskal-Wallis with Dunn’s post hoc test), although the differences were not significant relative to GL neurons (*p* = 0.0719; Kruskal-Wallis followed by Dunn’s post hoc test). In terms of GL neurons, primary and secondary dendrites were similar in length but significantly longer than the tertiary and quaternary dendrites (^*^ *p* < 0.05, **p < 0.01, ^***^ *p* < 0.001, ^****^ *p* < 0.0001; Kruskal-Wallis with Dunn’s post hoc test). There was also a significant difference in secondary dendrite length between GCL and GL neurons (^#^ *p* = 0.0326; Kruskal-Wallis with Dunn’s post hoc test). Intragroup variability didn’t show significant differences in total dendrite length **(F)** or in the number of branching points per neuron **(G)**. However, distinct branching patterns were observed between GCL and GL cells after performing a Sholl analysis **(H)**. GL neurons had more intersections 25–30 µm away from the soma, while GCL neurons presented a higher number of intersections 50–70 µm away from the cell body and they extended as far as 350 µm (^*^ *p* < 0.05; two-way ANOVA followed by Bonferroni’s post hoc test). The bars show the mean ± SEM: *n* = 28 (GCL), 4 (MCL), 3 (EPL), 42 (GL) cells **(B,D–G)**; *n* = 28 (GCL), 42 (GL) cells **(H)**. Scale bars in **(A)** = 10 μm **(C)** = 30 µm (confocal and projection images), 20 µm (isolated GCL neuron), 10 µm (isolated GL neuron). EPL, external plexiform layer; GCL, granule cell layer; GL, glomerular layer; IPL, internal plexiform layer; MCL, mitral cell layer.

The physical attributes of neurons are known to modify their electrophysiological properties. For instance, cell body volume is directly related to the cell’s capacitance, which influences synaptic efficacy (Gentet et al., 2000). However, no differences in cell body volume were evident between each cell body shape of CalR^+^ neuron (polygonal 188.52 ± 10.68 µm^3^, oval 227.01 ± 28.38 µm^3^, oval-polygonal 238.5 ± 33.99 µm^3^ and pear-shaped 213.65 ± 18.33 µm^3^). By contrast, neurons located in the GCL had larger somas than those in the GL (^****^ *p* < 0.0001; Kruskal-Wallis with Dunn’s post hoc test: Figures 3A,B). Therefore, we explored whether the specific layer to which the neurons are allocated affects their pattern of dendrite ramification and growth **(** Figure 3C). We analyzed the number and length of the neuronal processes and performed a Sholl analysis in order to further probe their characteristic morphologies (Figures 3D–H). The number of primary and secondary dendrites of cells located in the GCL was similar (Figure 3D), although the primary dendrites were significantly longer (^****^ *p* < 0.0001 vs. secondary, ^**^ *p* = 0.0010 vs. tertiary; Kruskal-Wallis followed by Dunn’s post hoc test) (Figure 3E). Moreover, these neurons had fewer tertiary dendrites (^****^ *p* < 0.0001 vs. primary, ^***^ *p* = 0.0003 vs. secondary; Kruskal-Wallis with Dunn’s post hoc test) and no quaternary dendrites (Figure 3D **)**. Conversely, GL interneurons had more secondary dendrites overall (^*^ *p* = 0.028 vs. tertiary, ^****^ *p* < 0.0001 vs. quaternary: Kruskal-Wallis with Dunn’s post-test) and significantly more tertiary dendrites than GCL neurons (^##^ *p* = 0.0014; Kruskal-Wallis with Dunn’s post-test). These neurons were also the only ones to possess quaternary dendrites (Figure 3D). Besides, their primary and secondary dendrites were similar in length, and they were significantly longer than the tertiary (^****^ *p* < 0.0001 vs. primary, ^***^ *p* = 0.0006 vs. secondary; Kruskal-Wallis with Dunn’s post-test) and quaternary processes (^**^ *p* = 0.0031 vs. primary, *p* = 0.0156 vs. secondary; Kruskal-Wallis followed by Dunn’s post hoc test: Figure 3E). No significant differences were found among the neurons located in the MCL and EPL.

Surprisingly, the difference in length between primary dendrites of GCL and GL neurons was not significant (*p* = 0.07; Kruskal-Wallis with Dunn’s post-test), whereas the secondary dendrites of cells in the GCL were significantly shorter (^#^ *p* = 0.0326; Kruskal-Wallis with Dunn’s post-test: Figure 3E). Moreover, no differences were detected between the neurons located in the different OB layers in terms of total dendrite length (Figure 3F) or the total number of branch points (Figure 3G), which probably reflected the high variability between cells in the same layer. Nevertheless, the Sholl analysis revealed changes in the dendritic arbor as a whole (Figure 3H), confirming differences in dendrite morphology between GCL and GL neurons. Although they don’t extend more than 80 μm, the dendrites of cells in the GL branched out more than those of GCL neurons, specifically at 25–30 µm from the soma (^*^ *p* < 0.05; two-way ANOVA followed by Bonferroni’s post hoc test). The dendrites of CalR^+^ neurons from the GCL covered larger distances, reaching as far as 350 μm, and with significantly more intersections 50 µm away from the cell body and beyond (^*^ *p* < 0.05; two-way ANOVA with Bonferroni’s post hoc test). Consequently, neurons from the GCL had dendrites that project over long distances, which may ramify once they reach their target. Meanwhile, the dendrites of GL cells branch more extensively, although their dendrites remain closer to the cell body. Together, these results suggest that the final layer localization of CalR^+^ neurons generated in the OB core may influence their acquisition of different dendritic morphologies.

### New CalR^+^ Interneuron Subtypes Identified in the Granule Cell Layer and Glomerular Layer

**FIGURE 4:**
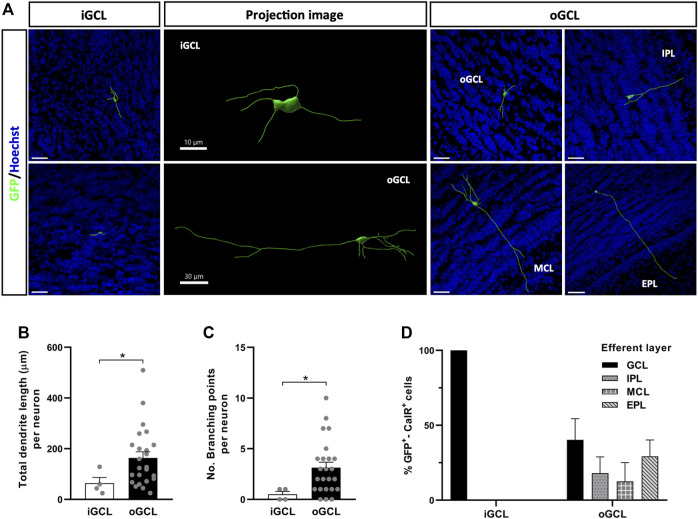
Different types of OB core-derived calretinin neurons are present in the GCL of the adult OB. **(A)** Representative 3D projections of neurons from the different portions of the GCL showing their distinct neuronal morphologies and the different layers they project to. **(B)** Neurons from the oGCL (86% of the total cells found in the GCL) had a larger total dendrite length (^*^ *p* = 0.048; Mann-Whitney U-test) and more branch points per neuron **(C)** relative to iGCL neurons (^*^ *p* = 0.027; Mann-Whitney U-test). **(D)** Dendrites of GCL neurons contact different layers, here defined as the efferent layer. All iGCL cells and 40% of the oGCL cells did not establish contacts with cells in other layers of the OB. Dendrites from the remaining 60% of the oGCL neurons were able to reach the EPL (29.17%), IPL (18.03%) or MCL (12.5%), with no significant differences in these percentages. The bars show the mean ± SEM: *n* = 4 (iGCL), 24 (oGCL) **(B,C)**; *n* = 4 (iGCL), 10, 5, 3, 6 (oGCL) cells **(D)**. Scale bars in **(A)** = 30 µm. EPL, external plexiform layer; GCL, granule cell layer; iGCL, internal granule cell layer; IPL, internal plexiform layer; MCL, mitral cell layer; oGCL, outer granule cell layer.

To identify CalR^+^ interneuron subtypes generated postnatally within the OB, we searched for differences between the cells located in the same layer. Due to the small number of GFP^+^-CalR^+^ neurons in the MCL and EPL, we focused on neurons located in the GCL and GL. Two neuronal subtypes were identified in the GCL based on their localization in the inner (iGCL) or outer (oGCL) portion of the GCL. This division was achieved on the basis of a higher accumulation of nuclei (as seen with Hoechst staining) and CalR^+^ cells in the oGCL than in the iGCL, which was clearly evident in coronal OB sections (Supplementary Figure S3). The iGCL interneurons accounted for 14% of the neurons in the GCL and they had simpler morphologies than the GCL cells that represented the remaining 86% of the neurons, as witnessed in the representative 3D projections (Figure 4A). Indeed, iGCL neurons had shorter total dendrite lengths than oGCL cells (^*^ *p* = 0.048; Mann-Whitney U-test) and fewer branch points (^*^ *p* = 0.027; Mann-Whitney U-test: Figures 4B,C **)**. Moreover, iGCL neurons only established local contacts within the GCL, whereas the dendrites of oGCL neurons projected to different layers of the OB such as the IPL, MCL or EPL (Figure 4D). Although 40% of the neuronal processes of the oGCL cells also remained within the GCL, 29.17% reached the EPL, 18.03% the IPL and 12.5% were seen to contact the MCL. Hence, by using both morphological and projection criteria, we were able to describe five major GCL CalR^+^ interneuron subtypes (Figure 4D).

**FIGURE 5:**
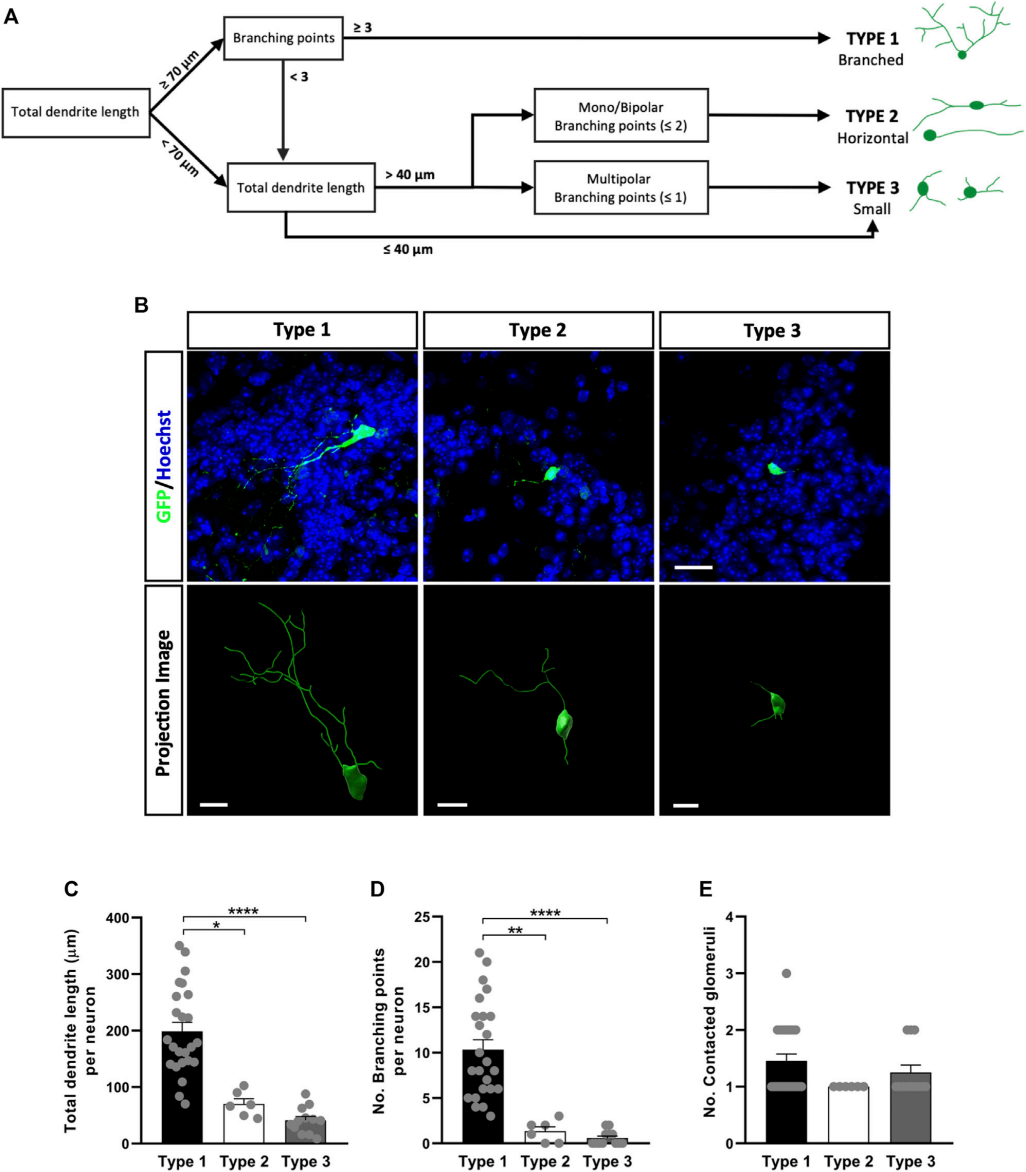
Different types of OB core-derived calretinin neurons are present in the GL of the adult OB. **(A)** Flow chart used to assign the glomerular neurons to different groups. According to their morphology, three subtypes were described: Type 1 (branched) GL cells with the longest and most ramified dendrites; Type 2 (horizontal) monopolar and bipolar cells with an intermediate pattern of dendrites in terms of length and ramifications; Type 3 (small) cells were the simplest, with short dendrites and few branching points. **(B)** Representative confocal microscopy images (top) of GFP^+^ neurons from each group or as three-dimensional projections (bottom). Total dendrite length **(C)** and the number of branch points **(D)** were analyzed to quantify differences in their morphology. As expected, type 1 cells had the longest total dendrite length (^*^ *p* = 0.0134 vs. type 2, ^****^ *p* < 0.0001 vs. type 3; Kruskal-Wallis with Dunn’s post hoc test) and they were more profusely ramified (^**^ *p* = 0.0024 vs. type 2, ^****^ *p* < 0.0001 vs. type 3; Kruskal-Wallis with Dunn’s post hoc test). **(E)** The number of glomeruli contacted by each neuron’s dendrites was counted but no significant differences were found between subtypes. The bars show the mean ± SEM: n = 24 (Type 1), 6 (Type 2), 12 (Type 3) cells **(C–E)**. Scale bars in **(B)** = 20 µm (upper images), 10 µm (lower images).

The neuronal subtypes in the GL were defined through the complexity and extent of their dendritic ramifications (Figures 5A,B): Type 1 GL neurons were those with a total dendrite length equal to or greater than 70 μm, and with three or more branch points, so we named these “Branched cells”; Type 2 cells had a dendrite length greater than 40 µm with only one or two primary processes (commonly extending in the same longitudinal axis) with less than three branch points, referred to as “Horizontal cells”; and finally, type 3 neurons were those with dendrites of a total length less than 40 µm and those with a total dendrite length more than 40 µm due to the presence of three or more very short primary processes, often unbranched, referred to as “Small cells”. These differences could be appreciated visually in confocal images and in projection images (Figure 5B). Indeed, our quantitative analysis showed that Type 1 neurons had the greatest total dendrite length (^*^ *p* = 0.0134 vs. Type 2, ^****^ *p* < 0.0001 vs. Type 3; Kruskal-Wallis followed by Dunn’s post hoc test: Figure 5C) and the largest number of branch points (^**^ *p* = 0.0024 vs. Type 2, ^****^ *p* < 0.0001 vs. Type 3; Kruskal-Wallis with Dunn’s post-test: Figure 5D). We also counted the number of glomeruli contacted by the dendrites of each neuron but no significant differences were found between these three subtypes of neurons. However, nine Type 1 neurons contacted two glomeruli and one neuron contacted three glomeruli, whereas only three Type 3 neurons contacted two glomeruli and all Type 2 neurons occupied just one glomerulus (Figure 5E).

### Tbr1 Overexpression Alters Neurogenesis in the Adult Olfactory Bulb

**FIGURE 6:**
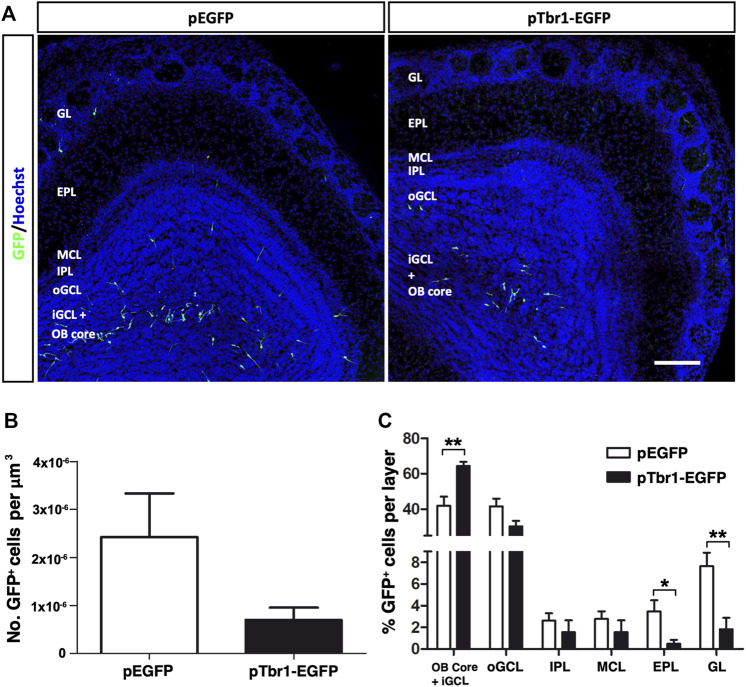
Tbr1 overexpression in the adult OB core affects the distribution of GFP^+^ cells across the different layers of the adult OB at 7 dpi. **(A)** Coronal sections of the OBs injected with the EGFP or Tbr1-EGFP particles showing distinct patterns of migration. **(B)** Fewer GFP^+^ cells were detected in the pTbr1-EGFP animals, although the difference wasn’t statistically significant. **(C)** Quantification of the GFP^+^ cells in each layer. The pTbr1-EGFP animals has a higher percentage of cells in the Core + iGCL (^**^ *p* = 0.0028; unpaired Student’s t test), yet a significantly lower proportion of neurons in the EPL (^*^ *p* = 0.0315; unpaired Student’s t test) and GL (^**^ *p* = 0.0051; unpaired Student’s t test). The bars show the mean ± SEM, n = 6 animals per condition. Scale bars in **(A)** = 150 µm.

We then asked whether adult OB neurogenesis could be regulated by transcription factor expression by injecting retroviral vectors expressing Tbr1, a transcription factor critical for the differentiation of mitral (glutamatergic) neurons in the OB (Bulfone et al., 1995; Méndez-Gómez et al., 2011; Docampo-Seara et al., 2019). We overexpressed Tbr1 in the adult OB core NSCs to examine whether this might induce the formation of mitral neurons, and/or modify the pattern of interneuron migration and differentiation. Most GFP^+^ cells expressed Tbr1 at 3 dpi when this construct was injected, whereas Tbr1 immunoreactivity was absent in GFP^+^ cells under control conditions (Supplementary Figure S2B). Increasing Tbr1 levels did not significantly change the total number of GFP^+^ cells at 7 dpi (Figure 6A) but it did affect the distribution of the GFP^+^ cells in the OB layers (Figure 6B). Following Tbr1 misexpression, a significantly higher proportion of cells were located in a region formed by the OB core plus the iGCL (^**^ *p* = 0.0028; unpaired Student’s t test), whereas this condition significant lowered the proportion of cells in both the EPL (^*^ *p* = 0.0315; unpaired Student’s t test) and GL (^**^ *p* = 0.0051; unpaired Student’s t test) relative to those expressing EGFP alone (Figure 6C).

**FIGURE 7:**
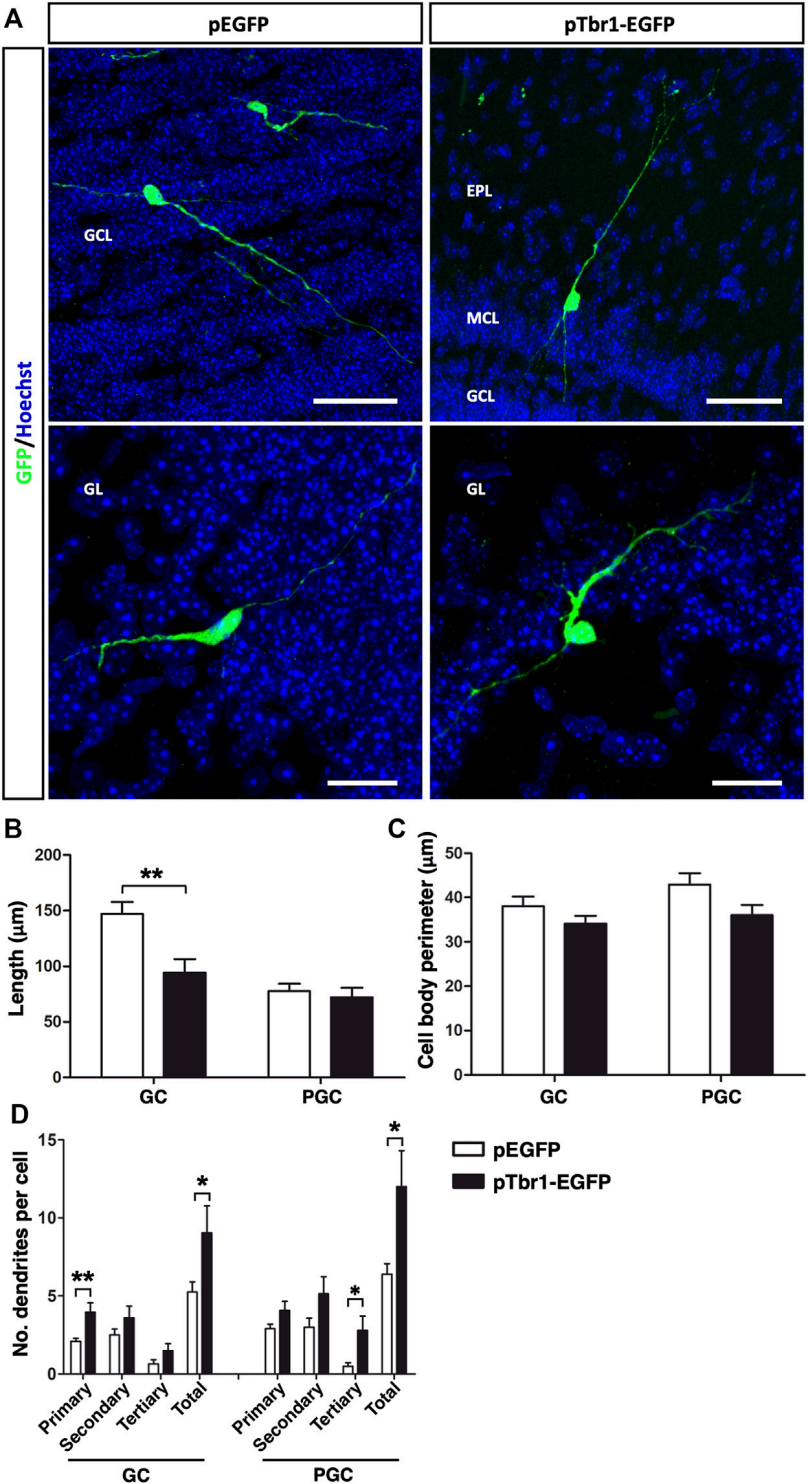
Tbr1 overexpression in the adult OB core influences the morphology of newly born GCs and PGCs. **(A)** Immunohistochemical staining for GFP showing the distinct morphologies acquired by GCs and PGCs in control (pEGFP) and Tbr1 overexpressing (pTbr1-EGFP) OBs. Quantification of these differences revealed a reduction in total dendrite length **(B)** on the GCs of pTbr1-EGFP animals (^**^ *p* < 0.01; unpaired Student’s t test), while the cell body perimeter **(C)** remained similar in both conditions. **(D)** The total number of dendrites was greater for both GCs and PGCs when Tbr1 was overexpressed (^*^ *p* < 0.05; unpaired Student’s t test). More specifically, there was an increase in the number of primary dendrites on the GCs (**p < 0.01; unpaired Student’s t test) and significantly more tertiary dendrites on PGCs (^*^ *p* < 0.05; unpaired Student’s t test) in pTbr1-EGFP animals than in their control counterparts. The bars show the mean ± SEM: *n* = 20 (GCL), 15 (GL) cells. Scale bars in **(A)** = 50 µm (upper images), 25 µm (lower images).

The stage of neuronal differentiation was then assessed based on morphological criteria (Figure 7) and Tbr1 overexpression caused a significant reduction in total dendrite length per GC (^**^ *p* < 0.01; unpaired Student’s t test) but not that per PGC (Figures 7A,B), without affecting the cell body perimeter (Figure 7C). It also increased the total number of dendrites per cell of both neuronal subtypes (^*^ *p* < 0.05; unpaired Student’s t test), although Tbr1 specifically influenced the number of primary dendrites on GCs (^**^ *p* < 0.01; unpaired Student’s t test) and tertiary dendrites on PGCs (^*^ *p* < 0.05; unpaired Student’s t test: Figure 7D). All these findings suggest that enhancing Tbr1 expression in local OB core NSCs affects the migration and the morphological differentiation of newly generated interneurons in the adult OB. Although Tbr1 is necessary for the maintenance of a glutamatergic neuronal phenotype (Schuurmans et al., 2004; Hevner et al., 2006; Méndez-Gómez et al., 2011), our results suggest that Tbr1 alone does not appear to induce the formation of mitral neurons from OB core NSCs *in vivo*.

## Discussion

The inhibitory activity of interneurons is crucial for the correct functioning of neuronal networks (Tremblay et al., 2016). The ratio of GABAergic interneurons to excitatory neurons in the neocortex is 1:5 (Markram et al., 2004), whereas in the OB it rises to 100:1 (Shepherd et al., 2004). Indeed, GAD65/67^+^ neurons represent 80% of the OB cells, with CalR^+^ cells the most abundant interneuron subpopulation in the adult OB (Parrish-Aungst et al., 2007). This is consistent with 11–40 times more neurons derived from OB core NSCs expressing CalR as opposed to CalB, PV, TH or Tbr2 (Defteralı et al., 2021). Here we report that adult born CalR^+^ interneurons can acquire distinctive dendrite morphologies that were used to identify five GC subtypes and three PGC subtypes in the OB in a thorough morphological analyses using three-dimensional visualization. Since the pattern of dendritic arborization determines the synaptic contacts that can be established by a neuron (Urbanska et al., 2008), the existence of diverse interneuron subtypes that establish contacts at different levels of the olfactory circuits can reflect the functional plasticity of the OB during adulthood (Wu et al., 2020).

One interesting finding is that almost 50% of the interneurons generated locally in the OB are PGCs, while it is widely accepted that the majority (around 90%) of cells derived from the SVZ end up in the GCL (Kaplan et al., 1985; Carleton et al., 2003; Kelsch et al., 2010). However, while neurogenesis in the SVZ diminishes with age (Enwere et al., 2004; Mandairon et al., 2011), the PGC subpopulations and their distribution remain stable (Mobley et al., 2013). This difference could be justified by the OB core NSCs generating a greater PGC to GC ratio relative to neurons derived from SVZ NSCs. Indeed, it has been proposed that OB core NSCs are more quiescent than SVZ NSCs *in vivo* (Moreno-Estellés et al., 2012), yet whether this difference might affect the PGC to GC ratio would require further study.

GCs can be classified based on the location of their cell bodies, although it is more useful to include the range of their dendritic ramifications among such criteria given the potential functional relevance of this parameter. For instance, GCs in deeper positions of the GCL make contact with mitral cells’ secondary dendrites in the deep portion of the EPL, while superficial GCs establish synapses with tufted cell dendrites from the superficial EPL (Nagayama et al., 2014). This indicates that these cells are engaged in parallel but distinct inhibitory activities in projection neurons. Following this principle, four novel subtypes of OB interneurons derived from the anterior ventral domain of the SVZ were described previously (Merkle et al., 2014). Here, we define five different GC subtypes according to the position of their soma (iGCL or oGCL) and the layer they project to (GCL, IPL, MCL or EPL). In addition to their distinct origin (OB core NSCs or those from the SVZ domains), the dendritic trees of the interneurons described here do not fit with those described previously for SVZ-derived interneurons (Merkle et al., 2014). Essentially, although OB core-derived cells could potentially establish synaptic contacts in the same areas as SVZ-derived interneurons their dendritic morphologies don’t appear to resemble each other in doing so. We believe that the different morphologies we observe do represent true interneuron subtypes and not different stages of maturation. Indeed, after 21 dpi markers of maturation like synapsin-I, VGAT and pCREB can be detected in newly generated OB core neurons that already establish and receive synapsis (Defteralı et al., 2021). This data correlates with the synaptic integration and activity of adult SVZ-born GCs seen from 15 dpi onwards (Petreanu and Alvarez-Buylla, 2002; Mizrahi, 2007; Whitman and Greer, 2007; Ravi et al., 2017).

In the GL there are SACs that establish interglomerular contacts and PGCs that form intraglomerular circuits between several (Type 1 PGCs) or a single (Type 2 PGCs) glomerulus (Kiyokage et al., 2010). Since the PGCs we described here are CalR^+^ and mainly contact only one glomerulus, they are likely to be considered Type 2 PGCs (Kosaka and Kosaka, 2007). Despite being a defined subpopulation, we appreciated some morphological differences among the cells in our study. Particularly, Type 1 (Branched) cells extended multiple dendrites over a large area of the glomerulus and they probably contact multiple cells constituting that glomerulus. On the other hand, Type 2 (Horizontal) cells only had one or two primary processes that didn’t extend much into the glomerulus, and Type 3 (Small) cells were constrained to small areas between the glomeruli, with short processes that were probably destined to establish local contacts. These subtle variations might have an impact on the way olfactory information is processed right after it is transmitted by olfactory sensory neurons.

Proliferation of NSCs (either in the SVZ or OB core), neuroblast migration and differentiation and the integration of adult-born cells into preexisting circuits are processes that can be regulated by extrinsic and intrinsic factors. Notably, adult neurogenesis regulators include growth factors like FGF-2, IGF-1, and EGF (Kuhn et al., 1997; Hurtado-Chong et al., 2009; Vergaño-Vera et al., 2009), olfactory learning and exposure to odorants (Kelsch et al., 2009; Gheusi and Lledo, 2014; Lepousez et al., 2014; Siopi et al., 2016; Denizet et al., 2017; Grelat et al., 2018; Bragado Alonso et al., 2019; Zhou et al., 2020), transcription factor codes and epigenetic mechanisms (Zhao et al., 2003; Curto et al., 2014; Fujiwara and Cave, 2016; Delgado et al., 2020), or the layers to which cells are allocated (as described here). For instance, VEGF inhibition impairs dendritogenesis of adult-born OB interneurons (Licht et al., 2010), and the morphology of the distal dendrites of newly born GCs are influenced dynamically by the activity of principal cells (Breton-Provencher et al., 2014). Therefore, the morphology acquired by neurons located in a specific OB layer probably reflects the actions of cell autonomous determinants, layer-specific factors and the influence of synaptic and odorant activity from outside areas. This fate can be redirected upon expression of specific transcription factors (Díaz-Guerra et al., 2013). In fact, we show that the ectopic overexpression (misexpression) of Tbr1 in the OB core alters the migration and morphological features of newborn interneurons, the latter in a cell type dependent manner. This indicates a fine regulation of the local OB neurogenesis.

The mammalian OB is a plastic structure that relies heavily on adult neurogenesis (Lazarini and Lledo, 2011), although some variation certainly exists between species. For instance, the cellular organization of the mouse SVZ-RMS differs from that of the human SVZ-RMS (Lim and Alvarez-Buylla, 2016), and neuroblasts migrate towards the OB throughout the lifespan of mice, in contrast to the sharp drop in migrating neurons in the first years of human life (Sanai et al., 2011). As NSCs have been isolated directly from adult human OB tissue and neurons have been generated from them (Pagano et al., 2000; Marei and Ahmed, 2013; Marei et al., 2018), an endogenous, quiescent NSC population may exist within the mammalian OB, although further research will be needed to confirm this. While the cellular and molecular processes defined in mice may or may not be the same in humans, defining these could help establish a framework for the potential development of cell therapies.

In summary, using precise imaging and morphological analyses we found that mouse OB core NSCs generate different types of granule and periglomerular cells, and that the morphology of these newly generated interneurons is largely affected by the layer in which they are allocated. Since the physiological role of adult neurogenesis is currently believed to be more closely related to brain plasticity than brain repair (Obernier et al., 2014), strategies that aim to reprogramme cells through transcription factor expression or neuronal replacement might help us to overcome the more restricted nature of adult NSCs. In order to achieve this goal, it is necessary to fully understand the mechanisms regulating NSC behavior and the diversity of the neurons generated in the adult brain.

## Data Availability

The raw data supporting the conclusions of this article will be made available by the authors, without undue reservation.
