# Supplementary figures and images for: Morphological Diversity of Calretinin Interneurons Generated From Adult Mouse Olfactory Bulb Core Neural Stem Cells
Source: Front Cell Dev Biol. 2022 Jun 29;10:932297. doi: 10.3389/fcell.2022.932297 (PMC9277347; doi:10.3389/fcell.2022.932297)

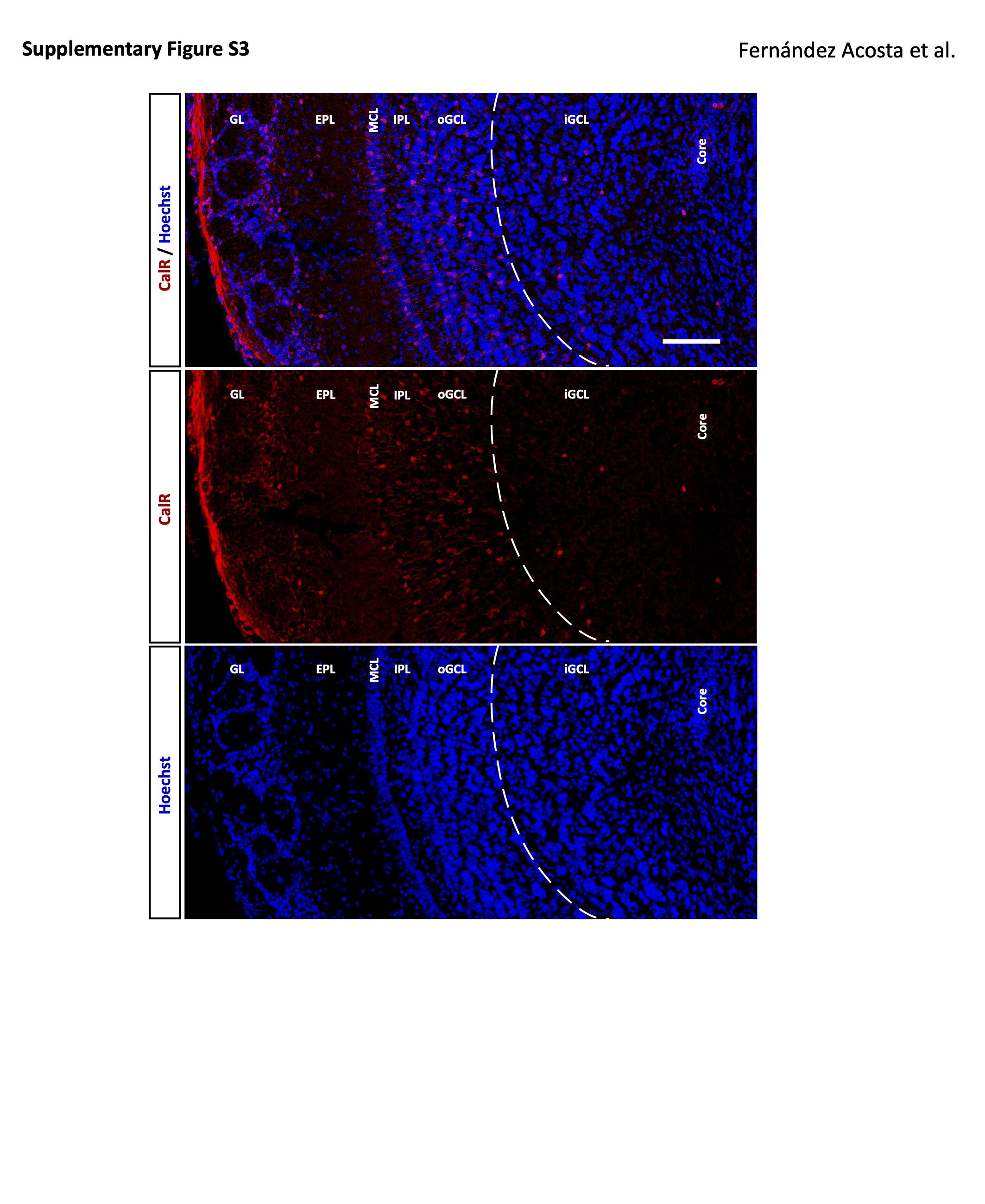

Supplement: Supplementary file 1 [file Image3.TIFF]

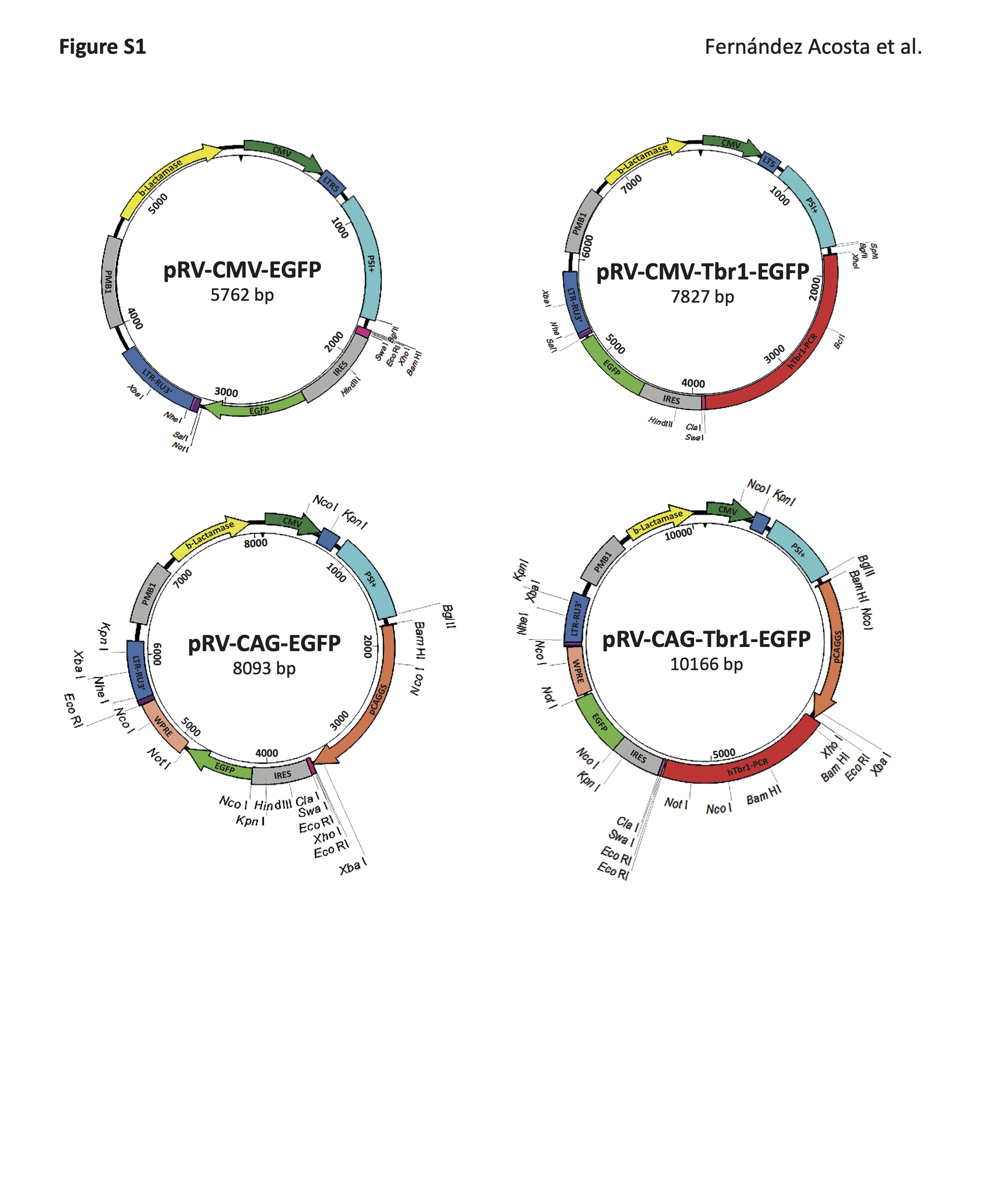

Supplement: Supplementary file 2 [file Image1.TIFF]

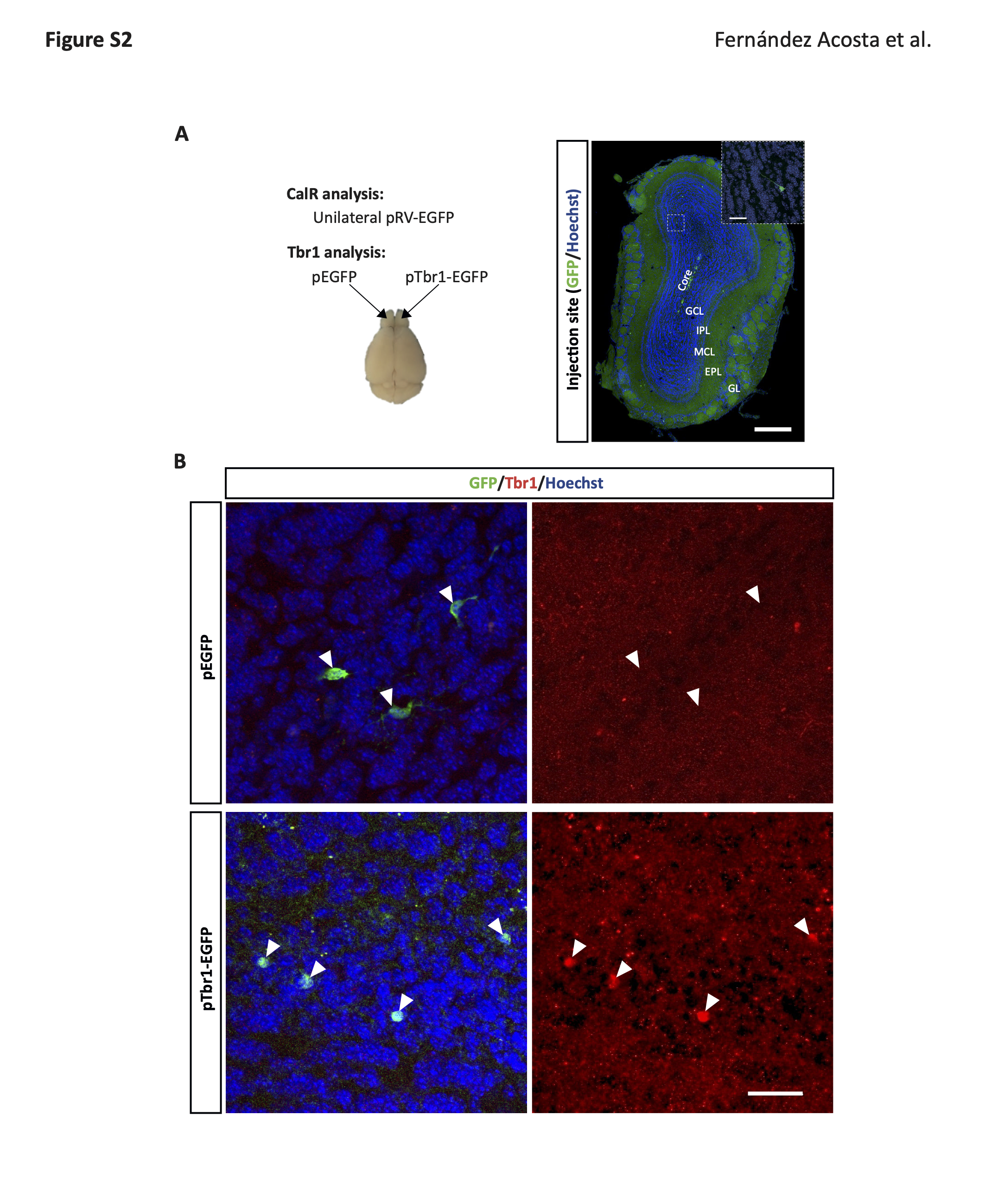

Supplement: Supplementary file 3 [file Image2.TIFF]
