# Supplementary material for: Morphological Diversity of Calretinin Interneurons Generated From Adult Mouse Olfactory Bulb Core Neural Stem Cells
Source: Front Cell Dev Biol. 2022 Jun 29;10:932297. doi: 10.3389/fcell.2022.932297 (PMC9277347; doi:10.3389/fcell.2022.932297)
Supplement: Supplementary file 5 [file DataSheet1.docx]

##### Morphological Diversity of Calretinin Interneurons Generated from Adult Mouse Olfactory Bulb Core Neural Stem Cells

**Figure S1**. Plasmid maps with the restriction sites used in our studies.

**Figure S2**. (**A**) Different retroviral particles were injected into each hemisphere, in line with the aim of our analyses. The accuracy of the injections can be assessed by the presence of GFP fluorescence in the OB core. The higher magnification image shows a newly generated GFP^+^ neuron derived from OB core NSCs. (**B**) At 3 dpi, animals injected with pTbr1-EGFP expressing particles show GFP and Tbr1 double labelled cells (arrowheads in lower panels), whereas animals injected with pEGFP expressing particles display single GFP expressing cells (arrowheads in top panels). Scale bars in **(A)** = 350 µm (inset = 30 µm) and in **(B)** = 30 µm.

**Figure S3**. The GCL can be divided into the inner (iGCL) and outer (oGCL) compartment, based on the abundance of CalR^+^ cells (middle image) and nuclei (Hoechst staining, bottom image) in the oGCL relative to the iGCL. Scale bar = 100 µm.

**Video S1**. Self-explanatory video illustrating the creation of the three-dimensional reconstructions of CalR interneurons derived from OB core NSCs.
